# Functionally refined encoding of threat memory by distinct populations of basal forebrain cholinergic projection neurons

**DOI:** 10.21203/rs.3.rs-3938016/v1

**Published:** 2024-02-09

**Authors:** Prithviraj Rajebhosale, Mala R. Ananth, Ronald Kim, Richard Crouse, Li Jiang, Gretchen López-Hernández, Chongbo Zhong, Christian Arty, Shaohua Wang, Alice Jone, Niraj S. Desai, Yulong Li, Marina R. Picciotto, Lorna W. Role, David A. Talmage

**Affiliations:** National Institutes of Health; National Institutes of Health; National Institutes of Health; Yale University; National Institutes of Health; Kansas City University of Medicine and Biosciences; National Institutes of Health; LinkedIn Corporation; National Institute of Environmental Health Sciences; Steris Corporation; National Institutes of Health; Peking University School of Life Sciences; Yale University; National Institutes of Health; National Institutes of Health

**Keywords:** acetylcholine, basal forebrain, amygdala, BLA, fear, anxiety, ventral pallidum, nucleus basalis, substantia innominata, cholinergic, modulation

## Abstract

Neurons of the basal forebrain nucleus basalis and posterior substantia innominata (NBM/SI_p_) comprise the major source of cholinergic input to the basolateral amygdala (BLA). Using a genetically-encoded acetylcholine (ACh) sensor in mice, we demonstrate that BLA-projecting cholinergic neurons can “learn” the association between a naïve tone and a foot shock (training) and release ACh in the BLA in response to the conditioned tone 24h later (recall). In the NBM/SI_p_ cholinergic neurons express the immediate early gene, Fos following both training and memory recall. Cholinergic neurons that express Fos following memory recall display increased intrinsic excitability. Chemogenetic silencing of these learning-activated cholinergic neurons prevents expression of the defensive behavior to the tone. In contrast, we show that NBM/SI_p_ cholinergic neurons are not activated by an innately threatening stimulus (predator odor). Instead, VP/SI_a_ cholinergic neurons are activated and contribute to defensive behaviors in response to predator odor, an innately threatening stimulus. Taken together, we find that distinct populations of cholinergic neurons are recruited to signal distinct aversive stimuli, demonstrating functionally refined organization of specific types of memory within the cholinergic basal forebrain of mice.

## Introduction

Acetylcholine (ACh) is critical for cognition. Basal forebrain cholinergic neurons (BFCNs), neurons that synthesize and release ACh that are sparsely distributed throughout the base of the forebrain, provide the primary source of acetylcholine to the cortex, hippocampus, and amygdala. Disruptions to normal cholinergic transmission are thought to contribute to several neuropsychiatric disorders (Sarter, Bruno and Turchi 1999, Higley and Picciotto 2014) as well as to cognition (Ananth, Rajebhosale et al. 2023) and salience-related behaviors (Jiang, Kundu et al. 2016, Hersman, Cushman et al. 2017, Crouse, Kim et al. 2020). BFCNs are anatomically divided into several clusters: the medial septum/diagonal band complex (MS/DB), the ventral pallidum (VP), the substantia innominata (SI) and the nucleus basalis (NBM). Between and within these anatomical groupings, BFCNs comprise heterogenous subclusters (Zaborszky, van den Pol and Gyengesi 2012). How this heterogeneity contributes to the significant control that cholinergic signaling exerts over large, behaviorally relevant circuits is unclear (Zaborszky, Csordas et al. 2015, Gielow and Zaborszky 2017).

Acetylcholine plays an important role in modulating emotionally salient memories (Luchicchi, Bloem et al. 2014, Ballinger, Ananth et al. 2016, Knox 2016, Ananth, Rajebhosale et al. 2023). We and others have found that cholinergic signaling in the basolateral amygdala (BLA) is important for generating defensive behaviors in response to both learned and innate threats (Power and McGaugh 2002, Jiang, Kundu et al. 2016, Wilson and Fadel 2017). Optogenetic manipulation of endogenous ACh release in the BLA during learning modulates the expression of threat response behaviors in mice upon recall of a conditioned stimulus (Jiang, Kundu et al. 2016). Stimulating release of ACh increases activity of BLA principal neurons, in part by increasing the release probability of glutamatergic inputs to these neurons, and is sufficient to induce long-term potentiation (LTP) when paired with minimal (non-LTP generating) stimulation of glutamatergic input to the BLA (Unal, Pare and Zaborszky 2015, Jiang, Kundu et al. 2016). Memory formation and retrieval are associated with fast synaptic mechanisms that are modulated by ACh, that are in turn necessary for the proper learning and expression of threat response behaviors (Nonaka, Toyoda et al. 2014). Given the broad distribution of cholinergic input across the BLA, and the well-established role of ACh in modulating BLA plasticity, the basal forebrain cholinergic system is well-positioned to serve an important role in the encoding of threat memories and generation of threat response behaviors (Ananth, Rajebhosale et al. 2023).

The BLA receives dense cholinergic input from neurons located in various regions within the basal forebrain (such as the VP, SI, and NBM). In this study we asked how these distinct populations of BLA-projecting BFCNs contribute to threat responses. Using a genetically encoded ACh sensor, activity-dependent genetic tagging, chemogenetic manipulations and electrophysiological recordings, we identify a population of BFCNs in the NBM/SI_p_ (SI_p_ defined as the portion of the sublenticular SI posterior to bregma − 0.4mm) that are required for learned threat responsiveness. We find that NBM/SI_p_ cholinergic neurons are necessary for freezing behavior following cue conditioned threat learning while freezing behavior elicited by an innately threatening stimulus activate cholinergic neurons in the VP/SI_a_ (VP/SI_a_; SI_a_ defined as the portion of the SI ventral to the anterior commissure located anterior to bregma − 0.4mm).

## Results

Animals recognize varied sensory stimuli and categorize them as either threatening or non-threatening. Recognition of threatening stimuli can be innate or acquired, for example, by association of an aversive experience with an innocuous, co-occurring sensory input. In this study we sought to understand if the basal forebrain cholinergic system participates in the encoding of associative threat or in response to innate threat.

### Acetylcholine is released in the basal lateral amygdala in response to threat.

**Figure 1:**
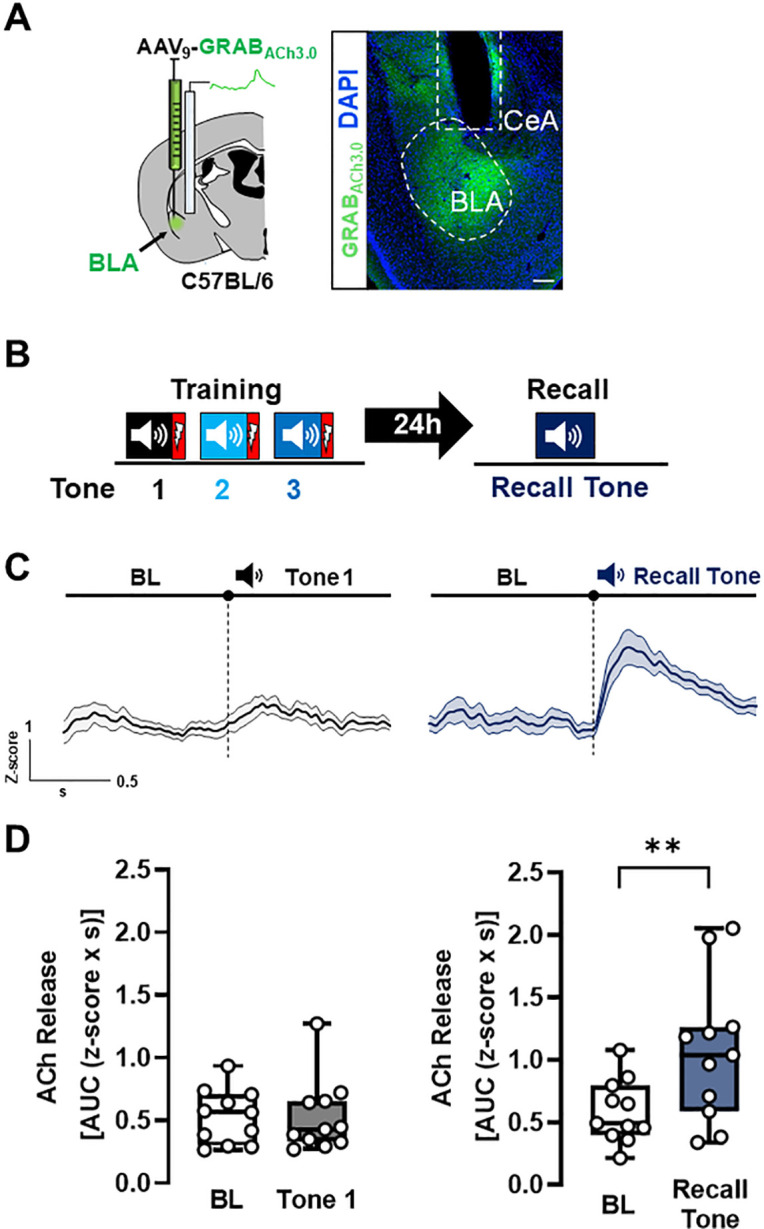
Acetylcholine is released in BLA during threat recall (see also Figure 1-Supplements 1–4). **A. Left**: Schematic of strategy for targeting the genetically encoded ACh sensor (GRAB_ACh3.0_) to BLA. **Right.** Image of ACh sensor expression (green). White dotted oval delineates ACh sensor labeled BLA. White dotted box denotes prior location of optical fiber. Scale bar = 100μm. Please refer to Figure S4 for fiber placement mapping in all mice. **B.** Schematic of the associative threat learning protocol employed consisting of 3 tone + shock pairings during the training period and tone alone during the recall session. **C.** Average traces of ACh release in response to tone; shading represents SEM: naïve tone (tone 1 during training) in black, recall tone in deep blue (tone onset indicated by bar above; n=11). BL=Baseline. **D.** Quantification of ACh release (area under the curve (AUC)) during baseline period (1s prior to tone onset) and in response to the first (naïve) tone and in response to the recall tone (1s following tone onset). Naïve tone did not induce significant increase in ACh release in the BLA (Wilcoxon matched-pairs signed rank test, BL vs. Tone1 p=0.8311. W=−6). Recall tone induced a significant increase in ACh release in the BLA (Paired t-test, BL vs. Recall tone p=0.0039 (two-tailed). t=3.732, df=10). See also Figures S1–4.

The BLA plays a central role in associative threat learning and in the generation of threat responses. We have previously demonstrated that silencing cholinergic input to the BLA during cue-conditioned threat learning (pairing a naïve tone with a foot shock) blunts learned freezing in response to the conditioned stimulus (tone) (Jiang, Kundu et al. 2016). Given this, the first question we asked was whether acetylcholine was released in the BLA during associative threat learning (Fig. 1 & Fig. 1-**Supplements1–4**). To monitor acute changes in extracellular ACh levels during the cue conditioned threat learning task, we expressed a genetically encoded ACh sensor, GRAB_ACh3.0_ (Jing, Zhang et al. 2018, Jing, Li et al. 2020) in BLA neurons and visualized fluorescence using fiber photometry (Fig. 1A). Our associative threat learning protocol involved placing mice in a novel chamber and exposing them to an 80dB tone for 30 sec. During the final 2 sec of the tone the mice received a foot-shock (0.7 mA). The tone-shock pairing was repeated twice (for a total of 3 pairings). Twenty-four hours later, mice were placed in a different chamber (with different tactile, visual, and olfactory cues to the training chamber) and exposed to tone alone.

Foot shock, either alone or paired with tone, increased ACh release in the BLA whereas the naïve tone i.e. the first tone before shock presentation (Tone 1), did not (Fig. 1-**Supplement 1C**, Fig. 1C & D, left; baseline (BL) vs. Tone 1, p = 0.8311). In contrast to Tone 1, the recall tone, presented twenty-four hours after the 3 tone-shock pairings, resulted in significant increase in ACh release in the BLA (Fig. 1C&D, right; p = 0.0039). The change in tone-associated ACh release required pairing with foot shock: naïve tone (Fig. 1-**Supplement 2C left,** p = 0.8437), three consecutive tones alone (without shock), or a subsequent repeat tone presentation after 24 hr (not previously paired with shock) (Fig. 1-**Supplement 2C right,** p = 0.3152), did not induce significant changes in ACh release in the BLA (Fig. 1-**Supplement 2)**.

To verify that the increases in ACh release were indeed specific to the tone-shock association and not due to generalization from prior shock exposure, we also subjected mice to 3 shocks (day 1) followed by a tone presentation 24 hr later (day 2) (Fig. 1-**Supplement 3A**). While mice demonstrated freezing behavior during the session on day 2, there was no significant increase in freezing behavior to the 24h tone presentation (Fig. 1-**Supplement 3C,** p = 0.2418). There was no increase in ACh in response to the tone when it was not explicitly paired with a shock, confirming that the changes in ACh release were indeed associative (Fig. 1-**Supplement 3D;** baseline (pre-tone, day 2) to 24h tone (tone presentation, day2): p = 0.7272). Therefore, after repeated tone-shock pairings, BLA projecting cholinergic neurons acquire enhanced tone responsiveness.

### NBM / SI_p_ cholinergic neurons are activated by threat learning and reactivated during threat memory recall.

**Figure 2:**
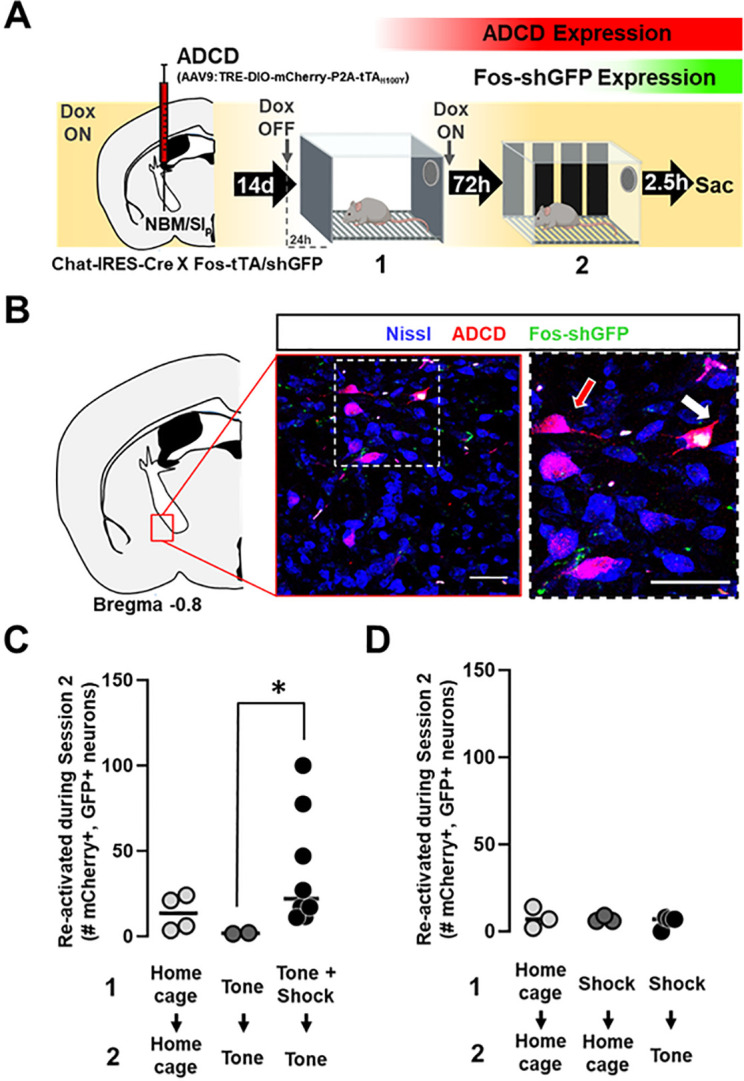
NBM/SI_p_ cholinergic neurons are activated by threat learning and reactivated during threat memory recall. (see Figure 2-Supplement 1). **A.** Strategy for labeling activated NBM/SI_p_ cholinergic neurons during both training and recall. Chat-IRES-Cre X Fos-tTA/shGFP mice (n=14) were injected in the NBM/SI_p_ with ADCD-mCherry virus (AAV9: TRE-DIO-mCherry-P2A-tTA_H100Y_). During Session 1 (off Dox) mice either remained in their home cage, were exposed to 3 tones (Tone alone), or were exposed to 3 tone-shock pairings (training session). During session 2, mice remained in home cage or were exposed to a single tone (recall session). Cholinergic neurons activated during training express ADCD-mCherry stably after training (red during training), and neurons activated during recall transiently express GFP (green during recall). **B.** Image of the NBM/SI_p_ showing cholinergic neurons activated during training (red arrow) or by both training and recall (reactivated – white arrow; image taken at A/P ~ −0.8 from Bregma; Scale bar = 50 μm). **C.** Quantification of the number of cholinergic neurons activated during Session 1 (ADCD-mCherry+) that were reactivated during Session 2 (both mCherry and GFP positive (activated both during Session 1 (training) and during Session 2 (recall)). Home cage (n=7 sections from 4 mice), tone only (n=4 sections from 2 mice) and tone + shock (n=17 sections from 8 mice) conditions. Significantly more cholinergic neurons were reactivated by tone following tone-shock pairings (Kruskal-Wallis p=0.0249). Tone-shock compared to tone only (p=0.0464, Dunn’s corrected). **D.** Quantification of number of reactivated cholinergic neurons (activated both during Session 1 and during Session 2 vs. the total number of cholinergic neurons activated during Session 1). Home cage→ome cage (n=9 sections from 3 mice), shock → home cage (n=17 sections from 4 mice) and shock → tone (n=11 sections from 3 mice). Kruskal-Wallis p=0.9471, KW=0.1219).

Following associative threat learning, cholinergic neurons exhibited increased ACh release in the BLA in response to a previously innocuous auditory stimulus; this increase occurred exclusively following pairing of the tone with a shock. Using a two-color labeling system, we asked whether NBM/SI_p_ cholinergic neurons were activated during the training session and reactivated during the recall session. To do this, we injected the offspring of a cross of Chat-IRES-Cre x Fos-tTA:Fos-shGFP with a viral vector, AAV_9_-TRE-DIO-mCherry-P2A-tTA^H100Y^, resulting in activity (tTA) dependent, Cre dependent (aka ADCD) mCherry expression (see methods and Fig. 2-**Supplement 1**). These mice carry three transgenes: one encoding Cre recombinase in cholinergic neurons, a second doxycycline (Dox) repressible, tetracycline transactivator (tTA) expressed following activation of the *fos* promoter, and a third destabilized green fluorescent protein (short half-life GFP) also under transcriptional regulation of the *fos* promoter. tTA and shGFP are transiently expressed in activated neurons. In the absence of Dox (delivered via chow diet), activation of Cre-expressing cholinergic neurons leads to tTA expression and expression of the virally transduced mCherry along with a mutant tTA, which is insensitive to Dox. Thus, after closure of the labeling window by re-administration of Dox, cholinergic neurons activated during the Dox off period maintain mCherry expression permanently driven by the mutant tTA. When ADCD labeling is coupled with the transient expression of Fos-shGFP, we can label and visualize participation of cholinergic neurons in two separate behavioral sessions (mCherry + = session 1 activated cells and GFP + = session 2 activated cells) (Fig. 2-**Supplement 1B**).

Two to three weeks following injection with the ADCD virus, mice were either (1) kept in home cage throughout, (2) exposed to tone without foot shock (tone alone), or (3) put through the standard threat learning paradigm (tone + shock). Twenty-four hours prior to the training session (session 1) mice were switched from Dox-containing to Dox-free chow to allow function of tTA. Immediately following tone-shock pairings, mice were placed back on Dox-containing chow (Fig. 2A). This switch from Dox on→Dox off→Dox on was also performed for mice that remained in their home cages and for those that were exposed to tones without shock. Recall was performed 72 hours later (tone alone in a new context), and mice were sacrificed ~ 2.5h following recall (the peak of the Fos-shGFP expression). We quantified the number of mCherry+/GFP+ (double positive) neurons following Session 2 (e.g. white arrow, Fig. 2B). Significantly more double positive cholinergic neurons were seen following the complete associative threat learning paradigm (tone + shock followed by tone recall) compared to mice that underwent session 1 without shocks (Fig. 2C, p = 0.0249). To further ensure that the reactivation of these cholinergic neurons was not due to a generalized increase in responsiveness of these neurons following shock exposure, we quantified reactivated neurons in mice exposed to shock alone during session 1 followed by tone alone during session 2 (shock alone (session 1) → tone alone (session 2)) along with shock alone (session 1) → home cage (session 2), and home cage controls (Fig. 2D). All three conditions showed few reactivated neurons and no differences between groups (p = 0.9471). Thus, associative threat learning results in activation of NBM/SI_p_ cholinergic neurons which are reactivated during subsequent cue-induced memory recall.

### Reactivation of cholinergic neurons activated by training is required for learned behavioral responses.

**Figure 3:**
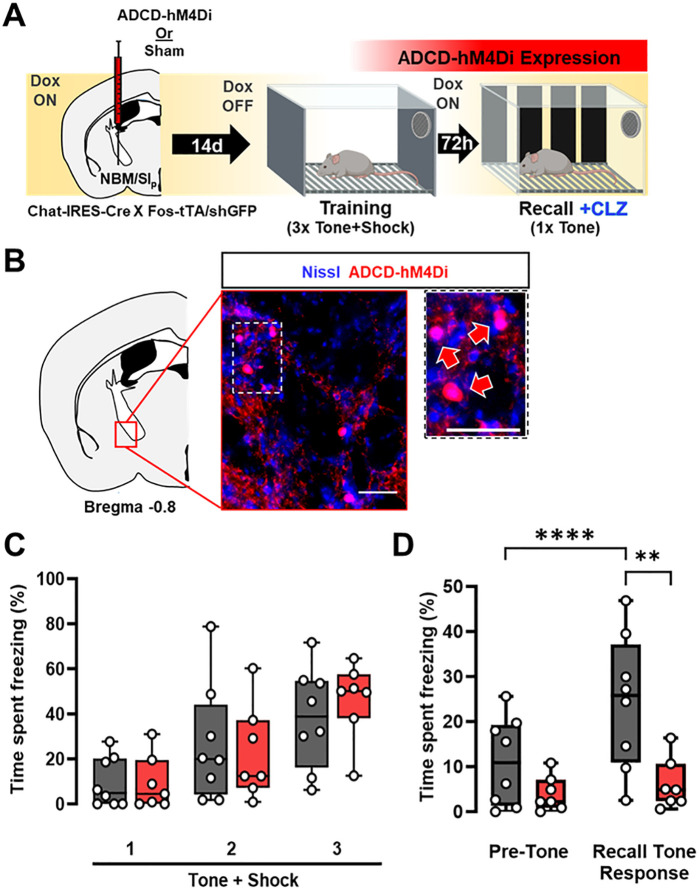
Re-activation of a subset of NBM/SI_p_ cholinergic neurons is required for threat memory retrieval. (see Figure 3-Supplement 1). **A.** ADCD-hM4Di (AAV9: TRE-DIO-hM4Di.mCherry) was injected into the NBM/SI_p_ of Chat-IRES-Cre x Fos-tTA/shGFP mice. Two weeks later mice underwent training on regular chow (Dox chow removed 24 hr prior to training session) to allow hM4Di.mCherry to be selectively expressed in training-activated cholinergic neurons. Three days later, recall was tested in Dox on conditions. Clozapine (CLZ) was injected 10min before the recall session to activate the inhibitory DREADD, hM4Di specifically in previously activated cholinergic neurons. **B.** Representative image taken at Bregma −0.8mm of mCherry (ADCD-hM4Di.mCherry) expressing cells. Inset shows higher magnification images of ADCD expression (red arrows). Scale bar = 50 μm. **C.** Freezing behavior during training in sham (grey, n=8 mice) and ADCD-hM4Di injected (red, n=7 mice) for each 30s bin during tone presentation (Tone + Shock 1, 2, 3). There were no significant differences between the groups during the training session (RM two-way ANOVA Time × Group p=0.6482; Group p=0.7311). **D.** Freezing behavior during recall following selective hM4Di mediated inhibition of training-activated cholinergic neurons in the NBM/SI_p_. Sham (grey, n= 8 mice) and hM4Di (red, n= 7 mice) groups. There were significant differences between pre-tone vs. tone-related freezing for sham (Pre-Tone vs. Recall Tone Response, p=0.0001, Bonferroni corrected), response to tone between sham and hM4Di (p=0.0026, Bonferroni corrected) and a significant main effect of Time × Group interaction (RM two-way ANOVA (GLM) Time x Group, p=0.0052). (See Figure 3-Supplement 1 for details on time periods comprising Pre-Tone and Recall Tone Response periods).

BLA-projecting cholinergic neurons acquire tone responsiveness following associative threat learning (Fig. 1) and a population of NBM/SI_p_ cholinergic neurons are activated during tone-shock pairing and reactivated during the recall session (Fig. 2). If these cholinergic neurons are indeed part of a threat memory engram, then their reactivation would be required for generation of learned threat responses. To block reactivation of cholinergic neurons in response to tone, we expressed the inhibitory, designer receptor hM4Di, in an activity dependent, Cre dependent manner in NBM/SI_p_ cholinergic neurons (ADCD-hM4Di; Fig. 3A) and subjected these mice to the threat learning paradigm (Fig. 3A). Mice were taken off Dox-chow 24 hours prior to the training session, immediately placed back on Dox-chow after training, and then tested for tone recall after 72 hr. ADCD-hM4Di and sham operated control mice were injected with clozapine (0.1 mg/kg; injected intraperitoneally (i.p.)) 10 min prior to the recall session to selectively silence the population of NBM/SI_p_ cholinergic neurons that were previously activated during training (Fig. 3A). Freezing behavior was quantified during both the training and recall sessions. Freezing was compared between the “Pre-Tone” period and “Recall Tone Response” (defined as freezing occurring from the onset of the recall tone through the end of the recall session) (Fig. 3-**Supplement 1B**). Both groups of mice showed the same freezing behavior during the training session (Fig. 3C, p = 0.6482. Figure 3-**Supplement 1A**). In the recall session, sham mice displayed typical freezing behavior in response to tone (Fig. 3D grey boxes; Pre-Tone vs. Recall Tone Response, p = 0.0001). In contrast, ADCD-hM4Di mice did not show increased freezing in response to the tone (Fig. 3D red boxes; BL vs. tone response, p = 0.8451). Overall ADCD-hM4Di mice showed lower freezing behavior compared to sham controls (Fig. 3D; sham – grey, hM4Di – red: p = 0.0052), indicating that reactivation of training-activated NBM/SI_p_ cholinergic neurons during the recall session was required for the expression of learned threat response behavior.

### BLA-projecting NBM / SI_p_ cholinergic neurons are reactivated during threat memory recall.

**Figure 4:**
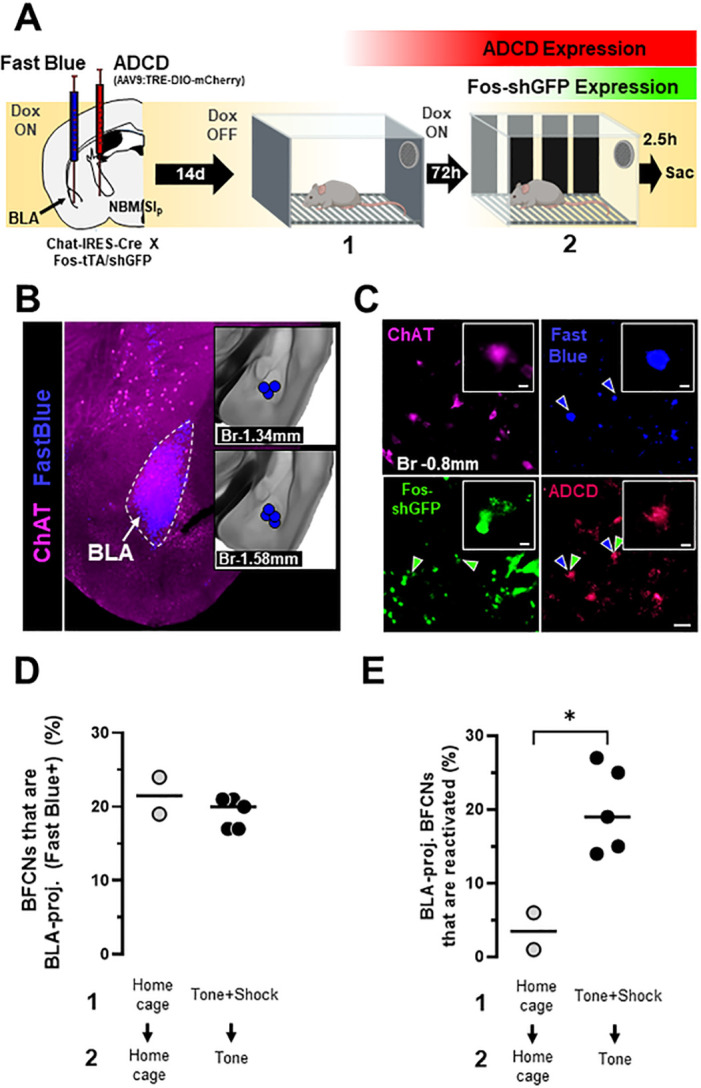
BLA-projecting NBM/SI_p_ cholinergic neurons are reactivated by the conditioned tone stimulus. **A. Left**, Strategy for labeling activated NBM/SI_p_ cholinergic during both training and recall along with mapping of BLA-projecting neurons. Chat-IRES-Cre X Fos-tTA/shGFP mice (n=7) were injected in the NBM/SI_p_ with ADCD-mCherry virus and in the BLA with Fast Blue dye. During session 1 (off Dox) mice either remained in their home cage or were exposed to 3 tone-shock pairings. During session 2 (recall session), mice remained in home cage or were exposed to a single tone. Cholinergic neurons activated during training express GFP transiently and express ADCD-mCherry stably after training (red during training), and neurons activated during recall transiently express GFP (green during recall). Neurons projecting to the BLA were labeled by Fast Blue (blue). Cholinergic neurons were identified by ChAT staining (magenta). **B.** Image of a Fast Blue injection site in the BLA; Inset: Mapping of injection sites for all Fast Blue experiments. **C.** Representative image showing (clockwise), ChAT+ neurons in the NBM/SI_p_ at bregma −0.8mm (magenta), BLA-projecting neurons (blue, blue arrowheads), training-activated cells (ADCD) (red) and recall-activated neurons (green, green arrow heads). BLA-projecting BFCNs activated by training and recall are denoted by double arrowheads (blue and green). Scale bar = 50 μm. Inset scale bar = 10 μm. **D.** Quantification of percentage of ChAT+ neurons that were labeled by Fast Blue in mice from the home cage group (grey) and mice from the training + recall group (black) from bregma −0.8mm. No significant differences were found between groups (Welch’s t-test, p=0.5192). **E.** Quantification of percentage of BLA-projecting BFCNs (ChAT+/Fast Blue+) at bregma −0.8mm that were reactivated during session 2 (ADCD+GFP) in mice from the home cage group (n=2) (grey) and mice from the training + recall group (n=5) (black). Mice that underwent training and recall had significantly higher number of engram-enrolled BLA-projecting BFCNs (Welch’s t-test, p=0.0183).

To investigate whether NBM/SI_p_ cholinergic neurons that are reactivated during recall are BLA-projecting, we injected Chat-IRES-Cre x Fos-tTA:Fos-shGFP mice with ADCD-mCherry in the NBM/SI_p_, and simultaneously delivered the retrograde tracer Fast Blue into the BLA (Fig. 4A). The mice were taken off doxycycline containing chow during the training period, returned to dox-chow for 72 hrs and then exposed to the tone alone. We then quantified BLA-projecting cholinergic neurons that were reactivated by tone (ChAT immunoreactive, Fast Blue labeled and ADCD-mCherry+/Fos-shGFP+; Fig. 4C). We found that ~ 20% of NBM/SI_p_ cholinergic neurons in both the home cage and threat-learning + recall paradigm group (at Bregma − 0.8mm) were labeled with Fast Blue, with no significant differences in the percentage of cholinergic neurons with retrograde label between groups (Fig. 4D; p = 0.5192). Next, we quantified the percentage of BLA-projecting NBM/SI_p_ cholinergic neurons that were active during session 1 and reactivated during session 2. We found that, on average, ~ 21% of BLA-projecting cholinergic neurons were reactivated during recall (Fig. 4E). This reactivation of BLA-projecting BFCNs was significantly higher in mice that underwent training + recall compared to mice that remained in their home cage but still underwent the Dox on→Dox off→Dox on protocol (Fig. 4E; p = 0.0183). Based on these data we conclude that BLA-projecting BFCNs are activated by associative threat learning and reactivated by threat recall.

### Silencing BLA-projecting basal forebrain cholinergic neurons during training or recall prevents activation of BLA neurons and conditioned freezing behavior.

**Figure 5:**
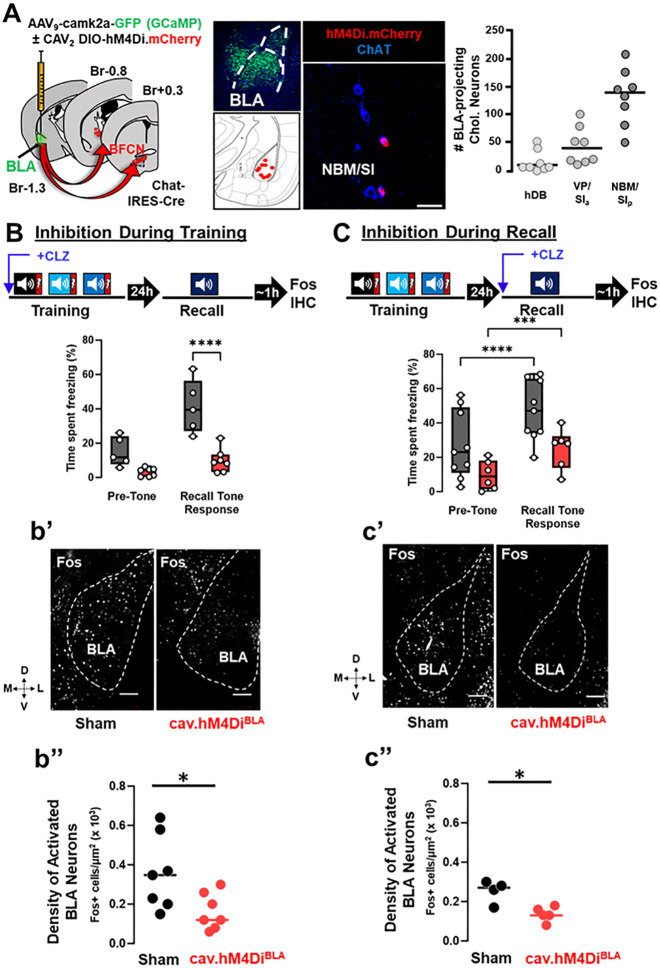
BLA-projecting cholinergic neuronal activity is required both during training and during recall for learned threat processing. (see Figure 5-Supplements 1 & 2) **A. Left.** Strategy for retrograde targeting of hM4Di DREADD to BLA-projecting cholinergic neurons. **Middle.** Re-localization of BLA injection sites (using AAV_9_-camk2a-GCaMP6f to mark the injection site), and identification of retrogradely-labeled cholinergic neurons within the NBM/SI_p_ (scale bar = 50μm). **Right.** Quantification of hM4Di-expressing cholinergic populations (mCherry+) across the basal forebrain (n=8 mice, 56–80 sections) (Bregma +0.6mm to −1.5mm). **B.** BLA-projecting cholinergic neurons were silenced by injecting mice with clozapine (CLZ) 10 min prior to training. Percent time freezing during the recall session including the pre-tone (baseline) period and in response to the conditioned tone. Clozapine was only administered during the training session. (RM Two-way ANOVA, Time x Group p=0.0047; Group p=0.0007). Sham vs. DREADD (Tone response, p<0.0001, Bonferroni corrected). **b’**, DREADD-induced silencing of BLA-projecting cholinergic neurons during training reduced BLA Fos immunoreactivity following recall. Representative BLA images from sham injected and CAV_2_-DIO-hM4Di mice fixed and stained with anti-Fos antibodies (white) at 45–60 min following recall. Dotted line outlines the BLA. (Scale bar = 100μm). **b”**, The density of recall-activated BLA neurons under sham injected conditions vs. following selective inhibition of the BLA projecting cholinergic neurons using CAV_2_-hM4Di (Fos+). Fos+ cell density in BLA sham injected (black) vs. CAV_2_-DIO-hM4Di.mcherry (red) (n= 7 mice/group, averaged from 22 sections sham vs. 28 sections hM4Di). Mann-Whitney test: p=0.0286. Lines represent median for each group. **C.** BLA-projecting cholinergic neurons were silenced during recall (clozapine given ONLY 10 min prior to the recall). Freezing differed significantly between pre-tone vs. recall tone response for sham and DREADD groups (RM two-way ANOVA, pre-tone vs. recall tone response, sham p<0.0001; DREADD p=0.0003). There was a significant effect of group, p=0.0312. sham and DREADD groups were significantly different in their response to the recall tone, p=0.0279. All multiple comparisons were Bonferroni corrected. **c’**, hM4Di-induced silencing of BLA-projecting cholinergic neurons during recall reduced BLA Fos immunoreactivity following recall. BLA images following Fos immunostaining. (Scale bar = 100μm). **c”**, Fos+ cell density in BLA between sham injected (black) vs. CAV_2_-DIO-hM4Di.mcherry (red) injected mice (n= 4–5 mice/group, averaged from 30 sections sham vs. 32 sections hM4Di). Mann-Whitney test: p=0.0317).

To determine whether chemogenetic silencing of BLA-projecting cholinergic neurons during training or during recall interfered with the activation of BLA neurons, we injected the BLA of Chat-IRES-Cre mice with CAV_2_-DIO-hM4Di.mCherry and AAV_9_-camk2a-GCaMP (cav.hM4Di^BLA^ mice) or AAV_9_-camk2a-GCaMP alone (sham mice) (Figs. 5A& Fig. 5-**Supplement 1A;** GFP fluorescence from GCaMP was used to mark the injection sites). We found mCherry was expressed in cholinergic neurons predominantly in the NBM/SI_p_, followed by the VP/SI_a_, with a small contribution from the horizontal limb of the diagonal band of Broca (hDB) (Fig. 5A right). These data support previous findings (Zaborszky, van den Pol and Gyengesi 2012) that NBM/SI_p_ cholinergic neurons provide a major input to the BLA.

We injected cav.hM4Di^BLA^ or sham control mice with clozapine (CLZ) 10 min prior to initiating cue-conditioned threat learning (Fig. 5B) or 10 min prior to the memory recall session (Fig. 5C). In both experiments mice were sacrificed 45–60 min following recall and assessed for Fos immunoreactivity (IR) in the BLA. We found that DREADD-mediated silencing of BLA-projecting cholinergic neurons during training alone blunted recall-induced freezing behavior and activation of BLA neurons (Fig. 5B: freezing behavior, sham vs. cav.hM4Di^BLA^ (Recall Tone Response), p < 0.0001, Fig. 5b’, b”: Fos density, sham vs. cav.hM4Di^BLA^ p = 0.0286). Similarly, DREADD-mediated silencing of BLA-projecting cholinergic neurons during recall alone also reduced recall-induced freezing and activation of BLA neurons (Fig. 5C: freezing behavior, sham vs. cav.hM4Di^BLA^ (Recall Tone Response) p = 0.0279, Fig. 5c’, c”: Fos density, sham vs. cav.hM4Di^BLA^ p = 0.0317). Mice in both sham groups showed equivalent freezing behavior (Fig. 5B & 5C, grey boxes; comparing sham groups, p = 0.8155) and density of Fos-IR cells (Fig. 5b’ & b”, black circles; comparing sham groups, p = 0.5273) indicating that 0.1mg/kg clozapine alone (in the absence of DREADD expression) did not alter Fos expression or expression of the learned threat response behavior. Thus, activity of BLA-projecting cholinergic neurons is required during both training and recall for recall induction of Fos expression in BLA neurons and freezing behavior. Preventing cholinergic neuron activity during either training or recall significant reduced the density of Fos + BLA neurons and tone-induced freezing.

Differences in recall-induced Fos expression between sham and cav.hM4Di^BLA^ mice were maximal in rostral portions of the BLA (between bregma − 0.8mm to − 1.4mm) (Fig. 5-**Supplement 1B**). This region of the rostral BLA has been shown to contain genetically distinguishable neurons that are activated by aversive stimuli and preferentially project to the capsular portion of the central amygdala (CeC), a region known to drive freezing behavior (Kim, Pignatelli et al. 2016, Kim, Zhang et al. 2017). We examined the CeC of mice in which BLA-projecting BFCNs were silenced during recall and found significantly reduced Fos + cell density in cav.hM4Di^BLA^ mice compared to control mice (Fig. 5-**Supplement 1C** sham vs. cav.hM4Di^BLA^ p = 0.0091). Thus, silencing cholinergic input to the BLA altered activation of BLA circuits involved in execution of defensive behaviors.

Mapping BLA-projecting BFCNs infected by CAV_2_-DIO-hM4Di revealed that the majority of the cholinergic input to the BLA originates in the NBM/SI_p_ (Fig. 5A). As such, we delivered AAV_9_-DIO-hM4Di.mCherry or AAV_9_-DIO-eCFP (sham mice) into the NBM/SI_p_ of Chat-IRES-Cre mice (Fig. 5-**Supplement 2**). Both hM4Di and eCFP animals were injected with clozapine 10 min prior to the recall session. Animals in which NBM/SI_p_ cholinergic neurons were silenced during the recall session did not show increased freezing in response to tone (Fig. 5-**Supplement 2A**, sham, grey boxes: Pre-Tone to Recall Tone Response, p = 0.0004; cav.hM4Di^NBM^, red boxes: Pre-Tone to Recall Tone Response, p > 0.9999). Thus, silencing NBM/SI_p_ BFCNs was sufficient to block expression of the learned threat response behavior.

### Recall-induced activation of NBM/SI_p_ cholinergic neurons correlates with the degree of threat response behavior.

**Figure 6:**
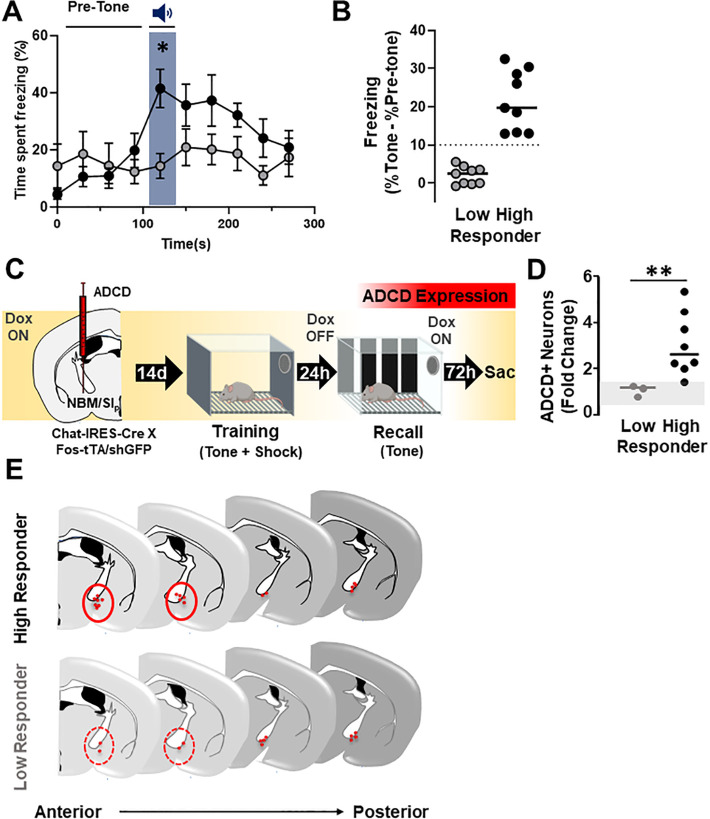
The extent of cholinergic neuronal activation in the anterior NBM/SI_p_ co-varies with the behavioral performance during threat-memory recall (see Figure 6-Supplements 1 & 2). **A.** Behavioral performance (freezing) from recall session showing High (black, n=9) and Low (grey, n=9) responding mice. High Responders show significantly higher freezing to recall tone whereas low responders do not. (Two-way RM ANOVA. Interaction effect (Time x Group classification, p=0.0001; Time, p=0.0042. High vs. Low Responder p=0.0454; Pre-tone vs. Tone: High Responder, p=0.0016; Low Responder, p>0.9999). All multiple comparisons were Bonferroni corrected. **B.** Quantification of change in freezing responses during recall session in Low and High Responders (pre-tone to tone). Dotted line delineates 10% points change in freezing, which was set as criteria for separating the two populations. (See Methods for rationale on stratification criteria. n=9 Low Responder, n=9 High Responder). **C.** Mice injected in the NBM/SI_p_ with ADCD-mCherry underwent training on Dox and recall off Dox to label recall activated NBM/SI_p_ cholinergic neurons (n=11). **D.** Quantification of change in number of cholinergic neurons activated (ADCD+) in low or High Responders relative to the home cage. The number of ADCD+ neurons differed significantly between Low and High Responders (Mann-Whitney test, p=0.01) (n=3 Low Responder, n=8 High Responder). Grey shading represents the range of fold-change in ADCD+ cells in individual home cage mice relative to the average of all home cage mice (n=5). (Mann-Whitney test, home cage v. Low Responder, p>0.9999; home cage v. High Responder, p=0.0121). **E.** Schematic showing anatomical distribution of ADCD-labeled NBM/SI_p_ BFCNs activated during recall across the anterior (bregma~ −0.8mm) to posterior (bregma~ −1.3mm) extent of the NBM/SI_p_ in High Responders (Top) vs. Low Responders (Bottom). Red circles highlight region of notable difference between High and Low responding mice.

During recall, we observed variability in individual freezing responses to the conditioned tone. Based on their responsiveness, we stratified the mice into two groups-high and low responders. ‘High Responders’ were defined as mice who showed a > 10 percentage points increase in time spent freezing in response to the tone compared to the pre-tone period (see methods for further details). Mice with < 10 percentage points increase in time spent freezing in response to the tone compared to the pre-tone period were defined as ‘Low Responders.’ When stratified as high or low responders according to this criterion, only High Responders showed a statistically significant increase in freezing during the recall tone compared to the pre-tone period (Fig. 6A; **Pre-tone vs. tone**: High Responders, p = 0.0016; Low Responders, p > 0.9999). High Responders showed more freezing compared to Low Responders specifically during the recall tone presentation (**High vs. Low responders**: recall tone blue shading, p = 0.0454). ‘High Responders’ spent more time freezing in response to the tone compared to the pre-tone period (Fig. 6B).

We next examined whether there was a relationship between the extent of freezing and the engagement of the cholinergic neurons. Since the majority of training-activated cholinergic neurons were reactivated during recall (in high responding mice - ~82% Fig. 6-**Supplement 1A**), we labeled cholinergic neurons activated during the recall session with ADCD-mCherry (on dox during training, off dox during recall; Fig. 6C). Next, we quantified the fold change in the number of mCherry + neurons in each group relative to corresponding home cage control mice (Fig. 6D). While there was no difference in mCherry expression in Low Responders compared to the home cage group (fold change ~ 1, p > 0.9999), High Responders displayed a 3-fold increase (p = 0.0121) in mCherry + cells (High Responders vs. Low Responders, p = 0.0121, Fig. 6D).

Mapping of recall-activated NBM/SI_p_ cholinergic neurons revealed that activated BFCNs in ‘High Responder’ mice were in anatomically distinct regions from those in ‘Low Responder’ mice (Fig. 6E). In a different cohort of “wild-type” mice, we assessed Fos and ChAT expression following recall and found that in the Low Responders, few ChAT and Fos co-labeled neurons were found. These colabeled cells were located in caudal regions of the NBM/SI_p_ (~ Bregma − 1.3; Fig. 6-**Supplement 2A-bottom row**). In High Responders an additional population of activated cholinergic neurons in more rostral portions of the NBM/SI_p_ was found (~ Bregma − 0.8; Fig. 6-**Supplement 2A- top row**). Thus, a discrete population of activated cholinergic neurons in the rostral NBM/SI_p_ is present in mice that respond to the learned threat. When comparing retrograde mapping of BLA-projecting cholinergic neurons using CAV_2_-DIO-hM4Di.mCherry (Fig. 5) to the distribution of ADCD-mCherry labeled activated neurons (Fig. 6), we find a similar distribution along the rostro-caudal axis of the NBM/SI_p_ (Fig. 6-**Supplement 2B&C**).

Finally, we examined the proportion of high and low responding mice in our experiments where we silenced BLA-projecting cholinergic neurons either during training or during recall (Fig. 5B & 5C). We found that under sham conditions (no cholinergic silencing), 80–90% of the mice were ‘High Responders”. Silencing BLA-projecting cholinergic neurons during training shifted the proportion such that 100% of the mice were ‘Low Responders’ (Fig. 6-**Supplement 1B** sham vs. cav.hM4Di^BLA^ inhibition during training). Silencing BLA-projecting cholinergic neurons during recall resulted in ~ 50% of the mice being ‘Low Responders’ (Fig. 6-**Supplement 1B** sham v. cav.hM4Di^BLA^ inhibition during recall). Thus, silencing BLA-projecting cholinergic neurons only during recall resulted in an all-or-none behavioral phenotype (50:50 chance of becoming a High or Low Responder).

### Cholinergic neurons activated during threat memory recall have altered intrinsic excitability.

**Figure 7:**
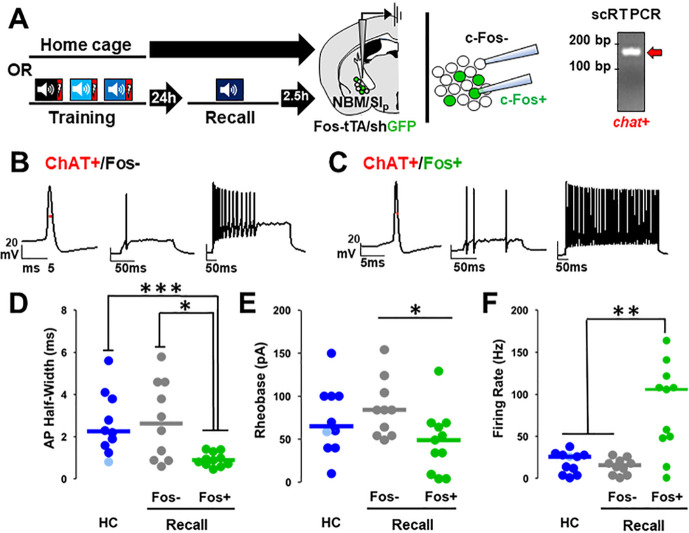
NBM/SI_p_ cholinergic neurons show increased intrinsic excitability following threat memory recall (see Figure 7-Supplement 1). **A.** Schematic of electrophysiological profiling of activated (Fos-shGFP+) vs. non-activated (Fos-shGFP−) neurons from mice following recall or in home cage mice, with *post-hoc* identification of cholinergic identity by single cell RT-PCR and evaluation of *chat* expression. **B.** Representative traces following injection of current into a Fos-shGFP− NBM/SI_p_ cholinergic neuron (ChAT+/Fos−). Red line denotes AP-half width measurement. **C.** Representative traces following step current injection in Fos-shGFP+ NBM/SI_p_ cholinergic neuron (ChAT+/Fos+). Red line denotes AP-half width measurement. **D, E, F:** Population data (dot plot + line at median) for the electrophysiological properties of *post-hoc* identified cholinergic neurons. Analyses assess passive and active membrane properties including action potential **(D)** (AP) half-width, **(E)** rheobase, and **(F)** maximal firing rate in response to 200–500 msec depolarization from rest potential (−60mV), from home cage (HC; n= 10–11 ChAT+ neurons from 10–11 mice) and following recall to tone alone (n= 10 ChAT+ Fos-shGFP− neurons from 5 mice vs. n= 11 ChAT+ Fos-shGFP+ neurons from 6 mice). **D**: Kruskal-Wallis tests; AP half-width: p=0.0054 (Dunn’s Corrected p-values: HC vs. Fos-shGFP−: p = 0.8971, HC vs. Fos-shGFP+: p = 0.0006, Fos-shGFP− vs. Fos-shGFP+: p = 0.0206) **E**: Rheobase: KW=p=0.05 (Dunn’s Corrected p-values: HC vs. Fos-shGFP−: p = 0.6153, HC vs. Fos-shGFP+: p = 0.0938, Fos-shGFP− vs. Fos-shGFP+: p = 0.0228) **F**: Max firing rate: p=0.0032 (Dunn’s Corrected p-values: HC vs. Fos-shGFP−: p = 0.3206, HC vs. Fos-shGFP+: p = 0.003, Fos-shGFP− vs. Fos-shGFP+: p = 0.0034).

Changes in excitability of neurons have been consistently associated with the threat memory engram (Zhang and Linden 2003, Zhou, Won et al. 2009, Cai, Aharoni et al. 2016, Rashid, Yan et al. 2016, Pignatelli, Ryan et al. 2019). We asked whether cholinergic neurons activated during memory recall differed in their intrinsic excitability compared to non-activated cholinergic neurons. To do this, we prepared acute brain slices from Fos-tTA/shGFP mice for electrophysiological recording of activated (Fos-GFP+) and non-activated (Fos-GFP−) NBM/SI_p_ neurons two and a half hours after the recall session or from mice that remained in their home cage. Cholinergic identity was verified post-recording by single cell RT-PCR of each recorded cell (Fig. 7A).

Cholinergic neurons that were Fos + following the recall session differed significantly from Fos cholinergic neurons (Fig. 7B&C) and from cholinergic neurons from homecage mice. Properties that showed significant differences included: action potential (AP) half-width, rheobase and maximum firing rate (Fig. 7D; half-width: HC vs. Fos-shGFP + p = 0.0006, Fos-shGFP− vs. Fos-shGFP + p = 0.021; Fig. 7E; rheobase: Fos-shGFP− vs. Fos-shGFP + p = 0.023; Fig. 7F; max firing rate: HC vs. Fos-shGFP + p = 0.003, Fos-shGFP− vs. Fos-shGFP + p = 0.0034) as well as latency to fire (Fig. 7-**Supplement 1E**; latency: HC vs. Fos-shGFP + p = 0.0062) and afterhyperpolarization (AHP) amplitude (Fig. 7-**Supplement 1F**, HC vs. Fos-shGFP + p = 0.0041). Resting membrane potential, AP amplitude, AP threshold, and AHP half-width did not differ (Fig. 7-**Supplement 1A-D**).

We also compared the firing rate of cholinergic neurons in home cage mice with those expressing Fos two and a half hours after training or at longer intervals following recall (measured 2.5 hr (Fos-shGFP) and at 3 and 5 days (ADCD labeling during recall) after the recall session Fig. 7-**Supplement 1G**). We found no differences in firing rate between home cage cholinergic neurons and cholinergic neurons that expressed Fos after training: that is the change in firing rate was only seen in cholinergic neurons activated during recall. This increase in maximal firing rate seen after recall returned to baseline within 3–5 d (compared to recall D0, p < 0.05 for all).

### Distinct subsets of BLA-projecting cholinergic neurons differentially contribute to learned vs. innate threat processing.

**Figure 8:**
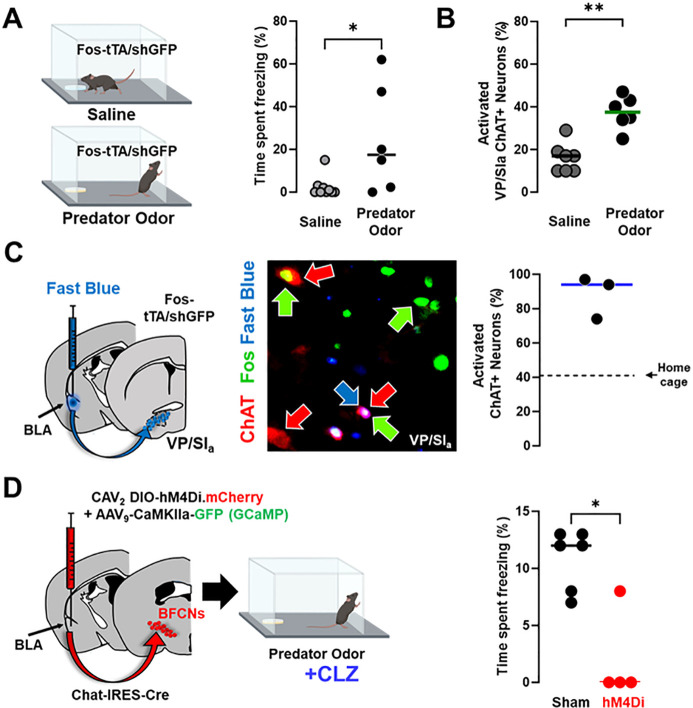
Distinct population of BLA-projecting cholinergic neurons contribute to innate threat processing (see Figure 8-Supplement 1). **A.** Fos-tTA/Fos-shGFP mice were placed in chambers containing a gauze pad spotted with either saline or with mountain lion urine (predator odor). Defensive behaviors were monitored for 5 min. Mice froze significantly more in the presence of predator odor than saline (Mann-Whitney, p= 0.028). **B.** Basal forebrain sections from the ventral pallidum (VP/SI_a_) of Fos-tTA/shGFPmice were immunostained for ChAT and GFP 45 min following odor exposure. Predator odor activated cholinergic neurons (GFP+/ChAT+) were quantified. Predator odor exposure increased the number of activated cholinergic neurons in the VP/SI_a_ (Mann-Whitney: p=0.0023), n=7 control and n=6 odor exposed mice. **C.** Fast Blue was injected into the BLA to retrogradely label BLA projecting neurons 6 days prior to odor exposure. After exposure to predator odor sections from the basal forebrain were immunostained with antibodies recognizing ChAT and Fos and the numbers of activated cholinergic neurons were counted (ChAT+Fos+/total ChAT+). In the VP/SI_a_ over 90% of BLA-projecting cholinergic neurons were activated (ChAT+ in red, Fos+ in green, Fast Blue in blue, n=3 mice). Dotted line indicates % of Fos+ cholinergic neurons in the home cage group in this experiment. **D.** Chat-IRES-Cre mice injected in the BLA with a control virus (AAV_9_-camk2a-GFP) alone (sham) or in combination with CAV_2_-DIO-hM4Di were exposed to predator odor following injection with clozapine (CLZ). Freezing behavior was measured during a 5 min exposure (scatter plot, bar indicates mean; sham – black, hM4Di – red). Silencing BLA-projecting cholinergic neurons significantly blunted the freezing response (Mann-Whitney: p=0.019; sham: n=6; hM4Di: n=4 mice).

Given the importance of BFCNs in a learned threat paradigm, we next asked whether these cells participate in innate threat responses as well. We stimulated an innate threat response by exposing Fos-tTA/shGFP mice to predator odor (mountain lion urine; Fig. 8A) (Blanchard and Blanchard 1990). Exposed mice increased active and passive defensive behaviors compared to mice exposed to a saline wetted pad, including freezing (Fig. 8A, p = 0.028), avoidance (Fig. 8-**Supplement 1B, left,** p = 0.0012) and defensive digging (Fig. 8-**Supplement 1B, right,** p = 0.023).

We quantified the number of cholinergic neurons expressing Fos (Fos-shGFP+) after saline or predator odor exposure (Fig. 8B/ Fig. 8-**Supplement 1A**; Fos-shGFP+/ChAT+). The number of Fos-shGFP expressing cholinergic neurons was significantly elevated in the predator odor exposed group in the VP/SI_a_ (Fig. 8B/ Fig. 8-**Supplement 1A-middle row**, p = 0.0023), but not NBM/SI_p_ (Fig. 8-**Supplement 1A-bottom row**, p = 0.4441) or the hDB (Fig. 8-**Supplement 1A-top row**, p = 0.2465).

VP/SI_a_ cholinergic neurons formed the second largest source of cholinergic input to the BLA in our retrograde mapping experiments (Fig. 5A). Since VP/SI_a_ cholinergic neurons were found to be activated during predator odor exposure, rather than NBM/SI_p_ or hDB cholinergic neurons, we asked if the BLA-projecting pool of VP/SI_a_ cholinergic neurons was activated by predator odor exposure. We injected the retrograde tracer Fast Blue into the BLA of Fos-tTA/shGFP mice and then exposed them to either saline (control) or predator odor (Fig. 8C left). Fast Blue labeled approximately 30% of ChAT-IR neurons located in the VP/SIa (data not shown). Nearly the entire subset of BLA-projecting VP/SI_a_ cholinergic neurons (median 94% ± Std.dev 12.5) were also GFP+ (Fig. 8C right).

To determine whether activity of these BLA-projecting cholinergic neurons was necessary for mice to freeze in response to predator odor, we used CAV_2_-DIO-hM4Di to silence BLA-projecting cholinergic neurons. Silencing during predator odor exposure resulted in significantly less freezing compared to sham mice (Fig. 8D, sham vs. cav.hM4Di^BLA^ p = 0.019). Other measures of active avoidance of the predator odor were not significantly altered by silencing BLA-projecting cholinergic neurons (Fig. 8-**Supplement 1C;** avoidance p = 0.8485; defensive digging p = 0.0714). These data support the conclusion that activity of BLA-projecting cholinergic neurons is critical for normal freezing behavior in response to innate threat. Taken together, we find that distinct populations of BLA-projecting BFCNs are involved in associative threat learning and the response to innately threatening stimuli.

## Discussion

A small number of sparsely distributed cholinergic neurons in the basal forebrain provide extensive innervation to most of the brain. These cholinergic neurons and their network of axonal terminal fields play a critical role in modulating cognitive processes (Ballinger, Ananth et al. 2016, Záborszky, Gombkoto et al. 2018).

To begin addressing whether the cholinergic system encodes stimulus-specific information, or whether it is generally recruited with salient experiences we monitored ACh release in the BLA during threat learning and retrieval. We anatomically mapped and electrophysiologically characterized behaviorally relevant BFCNs, and then investigated the contribution of different subsets of BFCNs to threat response behaviors. Taken together, our results demonstrate populations of cholinergic neurons that are an integral part of encoding a learned threat memory contribute to innate threat responses.

### Cholinergic Modulation of Associative Threat Learning

In the BLA, several molecular changes occur in response to learning CS-US associations, including new gene expression and protein synthesis (Sears, Schiff and LeDoux 2014). We used chemogenetics for projection-specific, cell-type specific silencing of cholinergic neurons. We used clozapine activation of hM4Di, acting at either cholinergic cell bodies and/or cholinergic terminals (Krashes, Koda et al. 2011, Ray, Corcoran et al. 2011, Ferguson, Phillips et al. 2013, Stachniak, Ghosh and Sternson 2014, Zhang, Jiang et al. 2017, Jin, Cheng et al. 2019, Nishioka, Hamaguchi et al. 2020, O’Neal, Nooney et al. 2020) to silence BLA-projecting BFCNs during training or during recall. The activation of hM4Di via systematically applied CLZ likely suppressed ACh release in the BLA via several mechanisms, acting both on the somata and/or nerve terminals of cholinergic neurons. This resulted in loss of freezing behavior as well as significantly reduced density of Fos expressing neurons in the BLA following recall (Fig. 5). This reduction of Fos expression in the BLA indicates that cholinergic signaling in the BLA contributes to appropriate BLA engagement during the acquisition and recall of threat memory. While our experiments did not directly measure the BLA engram *per se* (i.e. activation-reactivation of the same neurons within the BLA), our data support the hypothesis that BLA-projecting cholinergic neurons play a critical role in the formation and/or activation of the BLA engram.

We have previously demonstrated that activation of presynaptic acetylcholine receptors can induce sustained potentiation of glutamate release (McGehee, Heath et al. 1995, Zhong, Du et al. 2008, Jiang, Emmetsberger et al. 2013, Zhong, Talmage and Role 2013, Zhong, Talmage and Role 2015, Jiang, Kundu et al. 2016, Zhong, Akmentin et al. 2017). BLA neurons recruited during memory recall exhibit increased presynaptic glutamatergic activity (Nonaka, Toyoda et al. 2014). We further demonstrated that the increased glutamatergic transmission in BLA was dependent on presynaptic nicotinic acetylcholine receptors (nAChRs) located on glutamatergic terminals in the BLA, and that nAChR activation in the BLA was necessary for acquisition of conditioned threat memories (Jiang, Kundu et al. 2016). Based on these findings, we propose that chemogenetic silencing of BLA-projecting cholinergic neurons during threat learning or during recall results in loss of Fos expression due to alterations in presynaptic glutamatergic transmission resulting in disruption to the formation and/or recruitment of the BLA engram.

### Basal-Forebrain Cholinergic Neurons “Learn” to Respond to the Conditioned Stimulus.

In this study we used a genetically encoded ACh sensor (GRAB_ACH3.0_) to monitor endogenous ACh release in the BLA during threat learning and recall. First, we found that foot-shock rapidly and reliably evoked ACh release, in line with previous observations (Hangya, Ranade et al. 2015, Jing, Li et al. 2020). When we examined responses to the tone (CS, Fig. 1-**Supplement 1**), we did not detect a significant increase in ACh in the BLA in response to a naïve, unexpected tone. However, following conditioning, when mice were exposed to the conditioned tone in a novel environment 24h later, we observed robust ACh release in the BLA compared with the naïve tone (Fig. 1D). This enhancement of ACh release supports the notion that BLA-projecting BFCNs undergo physiological changes which allow robust responsiveness to previously naïve sensory stimuli. When mice were exposed to tones in the absence of footshocks and then exposed to the same tone 24h later, we did not detect increased ACh release in the BLA (Fig. 1-**Supplement 2**). Thus, plasticity of ACh release in the BLA in response to the tone requires pairing of the tone with a salient stimulus such as a footshock.

### Changes in excitability of Fos + cholinergic neurons

It has been proposed that alterations to synaptic weights and changes in ionic conductance resulting from learning-induced transcriptional programs allow for increased response fidelity during memory retrieval (Yap and Greenberg 2018). To assess whether such changes occurred in recruited cholinergic neurons following memory retrieval, we recorded properties of neuronal excitability from activated NBM/SI_p_ BFCNs (Fos+) and compared them with Fos-BFCNs recorded in the same brain slices (Fig. 7). Recall activated NBM/SI_p_ cholinergic neurons showed increased excitability which lasted for at least several hours following threat memory retrieval, returning to baseline within days. This finding is in line with previous reports of learning-associated changes in electrical properties, which are found shortly after recall, but disappear at later time points despite the persistence of the learned behavior (Moyer, Thompson and Disterhoft 1996, Pignatelli, Ryan et al. 2019). Observed changes in the electrophysiological properties were not present in Fos-shGFP + cholinergic neurons immediately following training, whose electrophysiological profiles were indistinguishable from cholinergic neurons in the home cage control group. Thus, many of the changes in electrical properties we observed were specific to recall-activated cholinergic neurons. Within recall-activated cholinergic neurons we find several changes consistent with an increased excitability such as decreased AP half-width, decreased rheobase, and an increase in maximum firing rate. Common features of activated neurons previously reported include similar increases in firing rate, with reductions in adaptation, decreased duration of post-burst afterhyperpolarization, decreased AHP amplitude, and synaptic alterations (Whitaker and Hope 2018).

### Differential contribution of distinct BLA-projecting BFCNs in learned vs. innate threat processing.

Amygdala microcircuits play an important role in the regulation of active vs. passive avoidance behaviors (Rickenbacher, Perry et al. 2017, Terburg, Scheggia et al. 2018). Our finding that silencing cholinergic input to the BLA resulted in a selective loss of threat-motivated freezing behavior supports potential specificity of cholinergic modulation within BLA microcircuits for freezing, but not active, defensive behaviors. We found that BLA-projecting cholinergic neurons were necessary for freezing in response to a learned threat-associated cue (Fig. 5), and for freezing in response to the innately threatening predator odor (Fig. 8). Direct silencing of NBM/SI_p_ cholinergic neurons attenuated learned threat induced freezing. Instead, predator odor activated BLA-projecting VP/SI_p_ cholinergic neurons and resulted in a freezing response. Based on these data, we propose that distinct populations of BLA-projecting BFCNs control freezing in response to fundamentally distinct threatening situations (learned vs. innate). Additionally, we note that while silencing BLA-projecting BFCNs did reduce freezing in response to predator odor exposure, it did not alter avoidance of the odor pad indicating that threat detection was still intact in these mice.

### Memory encoding in neuromodulatory systems.

Our study joins a growing literature demonstrating stimulus-encoding and rapid stimulus-contingent responses in various neuromodulatory neurons indicating that plasticity within subcortical modulatory circuits might represent a critical component of normal learning and memory recall. The BLA receives various modulatory inputs including dopamine (DA) from the VTA (Tang, Kochubey et al. 2020), noradrenaline (NA) from the locus coeruleus (LC) (Uematsu, Tan et al. 2017), and ACh from the basal forebrain. Including our present study, all three of these modulatory systems have been shown to be engaged during associative threat learning and retrieval. Each modulatory system seems to respond rapidly and robustly to aversive stimuli like mild electrical shocks, and activity within these systems during conditioning (i.e. during CS-US pairing) is critical for generation of freezing behavior during memory recall (Uematsu, Tan et al. 2017, Tang, Kochubey et al. 2020). VTA dopaminergic neurons have also been shown to display plasticity in tone-responsiveness such that a naïve tone does not result in significant firing of DA neurons (Tang, Kochubey et al. 2020). However, following 3 pairings of the tone with shocks, VTA DA neurons begin responding to tone presentations with millisecond latencies, a response that is sustained the following day during memory retrieval. A majority of the shock-responsive DA neurons were also found to acquire tone-responsiveness following pairing, a finding replicated within the cholinergic system in our study. While shock rapidly activates LC NA neurons, conditioned tone related responses in these neurons seems to be slow, occurring on average several seconds following tone presentation (Uematsu, Tan et al. 2017). How signaling by these different modulators interacts in the BLA and informs plasticity of BLA neurons is an intriguing question.

In addition to these modulators, peptides such as oxytocin have also been shown to participate in threat memory formation. A recent study demonstrated presence of a threat memory engram within the hypothalamic oxytocinergic projection to the amygdala (Hasan, Althammer et al. 2019). Interestingly, upon conditioning these neurons demonstrate a transmitter preference switch, releasing glutamate in the amygdala. Thus, subcortical neuromodulatory and peptidergic systems might display unique mechanisms of engram-related biophysical changes that have not been found in traditionally studied systems.

We demonstrate at least two populations of BLA-projecting cholinergic neurons that are engaged in learned vs. innate threat responses. Differences in function of other BLA-projecting BFCNs (NBM vs. HDB) in threat memory formation vs. extinction were recently demonstrated (Hasan, Althammer et al. 2019, Crimmins, Lingawi et al. 2023), further highlighting that effects of ACh release in the BLA are highly specific to which axons release the ACh, despite the dense overlapping terminal fields from different BFCN populuations within the BLA. Similar heterogeneity of responses has also been found in the dopaminergic and noradrenergic systems (Azcorra, Gaertner et al. 2023). It is possible that single cell transcriptomic analyses of the cholinergic basal forebrain may provide insight into the functional heterogeneity observed in our study.

### Is there a Cholinergic Component in the Associative Threat Memory Engram?

Studies examining mechanisms of learning and memory in recent years have revived Semon’s theory on memory engrams: learning must result in lasting biophysical changes that form the substrate for retrieval of the learned experience (Semon 1921, Tonegawa, Liu et al. 2015). Josselyn and Tonegawa have recently updated the definition of engram cells, requiring that these be activated by learning, modified by learning, and reactivated by subsequent presentation of the recall-inducing stimuli, resulting in memory retrieval (Josselyn and Tonegawa 2020). NBM/SI_p_ BFCNs investigated in this study indeed fulfil these criteria as they are activated by learning, show induction of Fos and altered physiological properties with recall, are reactivated by recall, and the reactivation of previously, training-activated BFCNs was necessary for recall behavior.

Multiple studies have used threat and reward learning paradigms in rodents to examine allocation of neurons to memory engrams. These studies have looked for these engram cells in regions such as cortex, amygdala and hippocampus focusing on glutamatergic pyramidal neurons (Josselyn, Köhler and Frankland 2015). However, recent work has demonstrated that memory engrams are distributed across brain-wide networks, and that reactivation of a multi-region engram more closely recapitulates natural recall behavior (Roy, Park et al. 2022).

In addition to the BLA, cholinergic neurons in the NBM/SI_p_ region project to various limbic and sensory regions such as the lateral orbital cortex, cingulate cortex, somatosensory cortex, and mediodorsal thalamus (Ananth, Rajebhosale et al. 2023). This raises the interesting possibility that the cholinergic signaling modulates various nodes of the threat memory engram circuit in conjunction with the amygdala, allowing for coordinated retrieval of engrams across distributed networks. Such coordinated activation of distributed engrams has been recently demonstrated to more closely recapitulate natural memory retrieval (Roy, Park et al. 2022). Furthermore, functionally related regions have been shown to receive their cholinergic input from the same cholinergic nucleus (Zaborszky, Csordas et al. 2015). We propose that engram-enrolled cholinergic neurons bind distributed engrams to encode stimulus-convergent, efficient memory retrieval.

## Materials & methods

### Resource availibility

#### Lead Contact

Further information and requests for resources and reagents should be directed to and will be fulfilled by Lead Contact, Dr. David Talmage (david.talmage@nih.gov).

#### Materials Availability

Plasmids generated in this study have been deposited to Addgene and will be available upon publication under Talmage Lab.

#### Data and Code Availabilit

This study did not generate/analyze datasets. Code for fiber photometry data was previously published in (Crouse, Kim et al. 2020).

### Experimental model and subject details

Adult (3–6 month) male and female Chat-IRES-Cre (B6;129S6-Chattm2(cre)Lowl/J, Jax stock number: 006410, (Rossi et al. 2011), Fos-tTA,Fos-shGFP (TetTag, Jax stock number: 018306, referred to as Fos-tTA/shGFP or Fos-shGFP), and Chat-IRES-Cre X Fos-tTA/shGFP mice were used. Mice within each cage were randomly assigned to experimental and control conditions. In all electrophysiology experiments, hemizygous Fos-tTA/shGFP mice on a C57BL/6 background were used. Mice were housed in a 12-hour light/dark cycle environment that was both temperature and humidity controlled. Mice had free access to food and water. All animal care and experimental procedures were approved by the Animal Care and Use Committees (ACUC) of the National Institute of Neurological Disorders & Stroke (NINDS) (Protocol #1531), SUNY Research Foundation at Stony Brook University (Protocol #1618), and Yale University (Protocol #2019–07895).

### Method details

#### Viral construct

##### Construction of the ADCD probe

All cloning unless otherwise specified was performed using In-Fusion HD (Clontech). “mCherry-P2A” was amplified using Phusion High-Fidelity DNA Polymerase (NEB) from pV2SGE (obtained as a gift from Dr. Shaoyu Ge Stony Brook University). “oChIEF-LoxP-Lox2272” was amplified from pV2.2 (synthesized gene block from IDT). The two fragments were cloned into pAAV-WPRE linearized by BamHI. The resulting plasmid was linearized by Pml I. “7xTetO-LoxP-Lox2272-tTAH100Y.SV40” was amplified from pV2.1 (synthesized gene block from IDT) and cloned into the Pml I site. The final plasmid was packaged into AAV_9_ viral particles. Viral packaging was performed by the University of Pennsylvania Vector Core.

##### Note re: ADCD expression in BLA neurons in the presence of doxycycline:

As shown in **Figure 2-Supplement 1C**, we noted “leaky” expression of ADCD-mCherry in the presence of doxycycline, in the BLA of Fos-tTA mice when co-injected with a Cre expression vector expressed from a camk2a promoter. Co-injection of camk2a-Cre and ADCD-mCherry into cortex and hippocampus of wild-type (C57) mice was also found to result in “leaky” expression despite the absence of genetically encoded tTA. Injection of ADCD-mCherry in hippocampus of PV-Cre mice did not result in expression similar to injection in Chat-IRES-Cre mice (**Figure 2-Supplement 1A,** bottom). These findings underscore the importance of performing the appropriate controls when using these vectors in vivo.

##### Construction of the ADCD-DREADD probe

“BglII-hM4Di.mCherry-AscI” was amplified using CloneAmpTM HiFi PCR Premix (Takara) from pAAV-hSyn-DIO-hM4D(Gi)-mCherry (Krashes MJ, et al. 2011) (gift from Dr.Bryan Roth; Addgene plasmid # 44362; http://n2t.net/addgene:44362; RRID:Addgene_44362). A backbone with TRE and Lox sites was ligated with “BglII-hM4Di.mCherry-AscI” using T4 DNA Ligase (NEB). The final plasmid was packaged into AAV_9_ viral particles. Viral packaging was performed by the University of North Carolina Vector Core.

##### Stereotaxic surgery & viral delivery:

Three-four-month-old ChAT-IRES-Cre mice were anesthetized and stereotaxically injected bilaterally. Coordinates were calculated based on the Paxinos Mouse Brain Atlas (Franklin, K & Paxinos, G, 1997): BLA (− 1.4mm A/P, ±3.5mm M/L, −4.8mm D/V), NBM (−0.7mm A/P, ±1.7mm M/L, −4mm D/V).

###### Tracers:

3% w/v solution of fast blue (FB) (17740–1, Polysciences Inc.) was prepared in sterile milliQ water. ~0.2μL of 3% FB was injected into the BLA bilaterally of Fos-GFP or Chat-IRES-Cre X Fos-tTA/shGFP mice. Mice were euthanized 7 days following injection.

##### Behavioral testing & analysis:

###### Threat conditioning:

All training and assessments were completed with experimenter blind to condition. Both training and recall sessions were analyzed using FreezeFrame v.3 (see below).

###### Habituation:

All mice were handled for a minimum of five minutes daily for three consecutive days before behavioral training began. For DREADD experiments, all mice were additionally habituated to restraint and injection with 100 μL saline administered i.p. daily.

###### Training:

On training day, all chambers were cleaned with 70% ethanol. Mice were placed into the behavioral chamber for a 10 min session which consisted of 3 min of habituation, followed by 3 tone-shock pairings (30 s 80dB, 5kHz tone, co-terminated with a 2 s 0.7mA foot shock with a 1.5 min interval between each pairing), and finally 2 min of exploration. For DREADD experiments, mice were given 0.1 mg/kg Clozapine (administered i.p.) (Sigma Aldrich) 10 minutes prior to being placed in the chamber.

###### Recall:

Recall session took place 24 – 72 hrs after completion of the training. To specifically test the response to tone-cued recall, the contextual features of the chambers were altered including texture of the floor, color of the walls, and scent of cleaner (mild lemongrass citrus-based solution). Mice were placed in the behavioral chamber for another 5 min session during which a single tone was delivered (30 s 80dB 5kHz tone) 2 min after being placed in the chamber. No shock was administered.

###### Analysis:

Percent time spent freezing was quantified using FreezeFrame v.3 (Actimetrics). Bout duration (defined as minimum required duration when animal is frozen) was set to 1 s, and threshold was manually defined as highest motion index with no movement other than breathing. Percent time spent freezing (defined as periods of no movement) was quantified across the 10 min session in bins of 30s. The following periods were defined for statistical analysis: Baseline (average of all bins prior to tone onset), Tone response (average of all bins following tone onset).

High Responders were defined as those mice that exhibited at least a 10-percentage point increase in % time spent freezing in the 30s bin during the tone from the average of the pre-tone period (e.g. Pre-tone freezing 10% to tone-induced freezing of ≥20%). All other mice were considered Low responders. Prior to any behavioral manipulation, mice showed up to 10% (of total time in given time bin) freezing indicating this level of freezing to be non-associative (potentially related to novelty or generalized fear). This criterion was found to give statistically significant difference between pre-tone vs. tone only for high-responders and not for low-responders providing further validity to the delineation of the Low and High Responder groups.

Analysis of population composition of High and Low responders (Figure 6-Supplement 1) was performed within experiment. Cross-experiment comparisons for population composition of High and Low responders was not possible due to differences in conditions and variability within and between cohorts.

###### Engram labeling:

Mice were placed on doxycycline hyclate-containing chow (Cat# TD.08541 Envigo) at least 2 days prior to injection of activity-dependent viral markers. Threat conditioning was performed as mentioned above. During doxycycline withdrawal, mice were transferred to a clean cage to prevent mice from eating dox food that was dragged into the cage or buried in the bedding. To minimize stress, some bedding containing fecal pellets and urine, and nest from the old cage were transferred to the new cage.

##### Predator odor exposure

###### Habituation:

All mice were habituated to restraint and injection with 100 μL saline administered i.p. daily for 3 days prior to behavioral testing for DREADD experiments. On exposure day, mice were transported to the lab several hours prior to exposure and habituated to the room and ambient sounds.

###### Exposure:

For exposure to predator odors, a vented mouse cage (L 13in × W 7.5in × H 5.5in) with corncob bedding (EnviroDri) was placed in a designated location in a laminar flow hood with overhead fluorescent lighting. Mt. Lion Pee (Maine outdoor solutions LLC) was obtained from predatorpee.com and stored at 4°C. 200μL of urine was pipetted onto a 3in × 3in 12 ply gauze pad (Cat#6312, Dukal corp.) placed in a polystyrene petri dish (VWR) at the vented end of the cage. Mice were placed into the cage in the end away from the odor and the cage was covered using a clear plexiglass barrier. Mice were exposed for 5 min and the session was filmed using an overhead digital camcorder (Sony). Following exposure, mice were returned to their home cage or a holding cage in the case of multiple housed mice to prevent any odor transfer. Control mice were exposed to 0.9% saline. For DREADD experiments, mice were given 0.1 mg/kg clozapine (administered i.p.; Sigma Aldrich) 15 minutes prior to being placed in the chamber.

Analysis: behavior was manually scored using Jwatcher (v0.9). Defensive digging was defined as vigorous digging performed by the mice using their snout, flinging bedding up and away from the animal. Freezing was defined as immobility without any obvious motion besides breathing. Cloth contacts were defined as front paw touches to the odor pad.

##### Fiber Photometry

###### Acquisition

Fiber photometry recordings were made using a Doric Lenses 1-site Fiber Photometry System. Signal was recorded using Doric Neuroscience Studio (V 5.3.3.4) via the Lock-In demodulation mode with sampling rate of 12.0 kS/s. Data was downsampled by a factor of 10 and saved as a comma-separated file. For details on connection of the setup refer to Crouse RB., et al. 2020.

###### Analysis

Preprocessing of the raw data was performed using a MATLAB script provided by Doric. The baseline fluorescence (F_0_) was calculated using a least mean squares regression over the duration of the recording session. The change in fluorescence for a given timepoint (ΔF) was calculated as the difference between it and F_0_, divided by F_0_, and multiplied by 100 to yield % ΔF/F_0_. The % ΔF/F_0_ was calculated independently for both the signal (465 nm) and reference (405 nm) channels and a final “corrected % ΔF/F_0_” was obtained by subtracting the reference % ΔF/F_0_ from the signal % ΔF/F_0_ at each timepoint. The corrected % ΔF/F_0_ was z-scored to give the final “Z % ΔF/F_0_” reported. Area under the curve was calculated for 1s duration before (baseline) and 1s after tone onset. The average of all the baseline periods within each analysis was used as the baseline reading for the AUC analysis.

##### Electrophysiology:

###### Brain slice preparation

For slice physiology, mice were anesthetized and transcardially perfused with cutting solution (sucrose 248 mM, KCl 2 mM, MgSO_4_ 3 mM, KH_2_PO_4_ 1.25 mM, NaHCO_3_ 26 mM, glucose 10 mM, sodium ascorbate 0.4 mM and sodium pyruvate 1 mM, bubbled with 95% O_2_ and 5% CO_2_) at 40°C. The brain was then rapidly removed and sliced, coronally, at 300 μM in oxygenated cutting solution at 40°C. Prior to recording, slices were incubated in oxygenated incubation solution (sucrose 110 mM, NaCl 60 mM, KCl 2.5 mM, MgCl_2_ 7 mM, NaH_2_PO_4_ 1.25 mM, NaHCO_3_ 25 mM, CaCl_2_ 0.5 mM, MgCl_2_ 2 mM, glucose 25 mM, sodium ascorbate 1.3 mM, and sodium pyruvate 0.6 mM) at room temperature.

###### Electrophysiological recording:

During recording, slices were superfused with oxygenated artificial cerebral spinal fluid (Jiang et al. 2016). Fos+ neurons were identified by GFP expression. Signals were recording using patch electrodes between 4–6 MΩ, a MultiClamp 700B amplifier, and pClamp10 software. Pipette internal solution was as follows: 125 mM K-gluconate, 3 mM KCl, 1 mM MgCl_2_, 10 mM HEPES, 0.2 mM CaCl_2_, 0.1 mM EGTA, 2 mM MgATP, and 0.2 mM NaGTP (pH = 7.3). Following recording, cytoplasm was harvested via aspiration for cell-type identification using single-cell RT-PCR. Ten-twelve basic electrical properties were determined and defined as previously described(López-Hernández, Ananth et al. 2017). Recordings were excluded if they did not meet the following criteria: 1. membrane potential less than or equal to −50 mV, 2. input resistance between 100–300 MΩ, 3. series resistance < 10 MΩ that was unchanged throughout the recording, and 4. firing a 45 mV action potential at rheobase

###### Single cell reverse transcription-PCR:

Single cell samples were pressure ejected into a fresh RT buffer prep (Applied biosystems). Samples were sonicated in a total volume of 20 μL at 40°C for 10 min before addition of RT enzyme mix (Applied Biosystem). Tubes were incubated at 37°C for 60 minutes and then 95°C for 5 minutes. Two rounds of amplification (30 cycles each) were done for the detection of Chat transcripts. For the first round of amplification (reaction volume 25 μL) included 2X mastermix, sterile water, 0.2 mM of each primer, 1 mL of cDNA sample). For the second amplification, the reaction included 1 μL of the previous (first-round) PCR product, 2X mastermix, sterile water, and 0.2 mM of each primer. Whole brain cDNA was run in parallel with the single cell samples. After amplification, the PCR products (159 bp) were analyzed on 3% gels.

##### Immunohistochemistry:

Following perfusion, brains were fixed overnight at 4°C in 4% PFA (in 1XPBS) and were then transferred to a 30% sucrose solution (in 1XPBS). Brains were flash frozen in OCT Compound (Tissue Tek) and stored at −80°C until cryosectioning. 50 μm cryosections were mounted onto Superfrost slides (Fisher Scientific) in sets of 3 and allowed to dry overnight at room temperature. Sections were blocked overnight at 4°C in a PBS solution containing 0.3% TritonX-100 and 3% normal donkey serum and then incubated with primary antibody in a PBS-T solution (0.1% TritonX-100 and 1% normal donkey serum), overnight (24h at 4°C). The next day, sections were rinsed in PBS-T and incubated in secondary antibody for 2 hr at room temperature in PBS-T along with NeuroTrace-435 (Invitrogen). Sections were treated with an autofluorescence eliminator reagent (EMD Millipore) according to the manufacturer’s guidelines and mounted in Fluoromount-G (Southern Biotech). Details regarding antibodies can be found in the Key Resources Table (KRT).

### Quantification and statistical analysis

#### Imaging and analysis:

All imaging was conducted on an Olympus wide-field slide-scanner microscope at 20X magnification (VS-120 and VS-200 systems, Z-step= 3 μm). Images were processed using the cell counter plugin on ImageJ. For Fos+ cell counts in the amygdala, only neurons (Nissl/ Neurotrace positive) with nuclear Fos stain were counted. The amygdala was identified, and a region of interest (ROI) defined using ROI manager in Image J. Total area of the ROI was measured and noted. Fluorescence threshold was set to eliminate background fluorescence in ImageJ (defined as hazy background signal detected in space between neurons and white matter). This eliminated non-specific fluorescence and out of focus signals. Fos+ nuclei were then counted using the cell counter plugin.

For ADCD cell counts, mCherry+ neurons at the NBM/SI injection site were counted. NBM was consistently identified as the cluster of cholinergic cell bodies at the base of the internal capsule in the Globus Pallidus and the SI as the area located directly ventral to the GP as denoted by the Paxinos Mouse Brain Atlas (3^rd^ Edition). 100% of the analyzed area of every third brain section was counted (~150 μm apart). Since the NBM/SI regions lack defined boundaries, we present the data as cell counts as opposed to cell density.

For Fos analysis in the BLA, Fos+ cells were counted in the area enclosed within the external and amygdalar capsules. Since the shape of the BLA changes along the anterior-posterior axis, Fos+ cell counts were normalized to the area enclosed within the external and amygdalar capsules and presented as density of Fos+ cells.

### Statistical analysis:

Statistical analyses were done using GraphPad Prism (GraphPad Software Inc., San Diego, CA, USA), Sigmaplot 12.5 (Systat Software, Inc., San Jose, CA, USA) and OriginPro 9.1 (Origin Lab Corporation, Northampton, MA, USA). Normality of the data was assessed using Shapiro-Wilk and Smirnov-Kolmogorov tests. Data that were not normally distributed according to both normality tests, were analyzed using appropriate non-parametric tests. Detailed information on statistical tests used, p-values, and sample sizes, and other descriptive statistics can be found in the text (Figure Legends) and/or in the statistical reporting table (Supplementary File 1). Sample sizes for behavior experiments were determined using a power calculation based on effect sizes in pilot experiments with power set to 0.8.

Parametric tests used: Repeated Measures (RM) One-way ANOVA, RM Two-way ANOVA, Welch’s ANOVA, paired t-test (two tailed), Welch’s t-test.

Non-parametric tests used: Mann-Whitney test, Wilcoxon matched-pairs signed rank test, Kruskal-Wallis Test, Friedman Test.

p-value criteria: * p≤0.05, ** p≤0.01, *** p≤0.001, **** p≤0.0001

## Figures and Tables

**Key Resources Table:** 

Reagent type (species) or resource	Designation	Source or reference	Identifiers	Additional information
strain, strain background (*Mus musculus*)	Chat-IRES-Cre	The Jackson Laboratory	B6;129S6-Chattm2(cre) Lowl/J	stock number: 006410
strain, strain background (*Mus museulus*)	Fos-tTA,Fos-shGFP	The Jackson Laboratory	TetTag	stock number: 018306
strain, strain background (*Escherichia coli*)	Stellar Competent Cells, HST08	Takara	Cat#636766	
genetic reagent (*AAV9*)	AAV9-camk2a-GCaMP6f-WPRE-SV40	U. Penn Vector Core		
genetic reagent (*AAV9*)	AAV9-DI0-eCFP	This paper, Vector Biolabs		Custom made
genetic reagent (*AAV9*)	AAV9-hSyn-GACh4.3	Vigene Biosciences Inc.		
genetic reagent (*AAV8*)	AAV8-DI0-hM4Di-mCherry	Addgene	Cat#44362	
genetic reagent (*AAV9*)	AAV9-TRE-DI0-oChlEF-mCherry-P2A-tTAH100Y.SV40	This paper	Cat#169414	Deposited to Addgene
genetic reagent (*AAV9*)	AAV9-TRE-DI0-hM4Di-mCherry	This paper	Cat#169415	Deposited to Addgene
genetic reagent (*CAV2*)	CAV2-DI0-hM4Di-mCherry	Dr.EJ Kremer, Institut de Génétique Moléculaire de Montpellier, France.		
antibody	anti-ChAT (Goat polyclonal)	Millipore	Cat# AB144R RRID:AB_2079751	IHC (1:500)
antibody	anti-GFP (Rabbit polyclonal)	Thermo Fisher Scientific	Cat#: A-11122; RRID:AB_221 569	IHC (1:1000)
antibody	anti-GFP (Rabbit polyclonal)	Abeam	Cat#: ab13970; RRID:AB_300798	IHC (1:500)
antibody	anti-mCherry (Mouse monoclonal)	Takara	Cat#: 632543; RRID:AB_2307319	IHC (1:500)
antibody	anti-DsRed (Rabbit polyclonal)	Takara	Cat#: 632496; RRID:AB_10013483	IHC (1:500)
antibody	anti-c-Fos (Rabbit polyclonal)	Synaptic Systems	Cat#: 226003; RRID:AB_2231974	IHC (1:500)
antibody	anti-Rabbit IgG (H + L)-AlexaFluor 488 (Donkey polyclonal)	Thermo Fisher	Cat#: A32790; RRID:AB_2762833	IHC (1:1000)
antibody	anti-Rabbit IgG (H + L)-Rhodamine Red-X (Donkey polyclonal)	Jackson Immunoresearch	Cat#: 711–295-152; RRID:AB_2340613	IHC (1:1000)
antibody	anti-Goat IgG (H + L)-AlexaFluor 594(Donkey polyclonal)	Thermo Fisher	Cat#: A-11058; RRID:AB_142540	IHC (1:1000)
antibody	anti-Chicken lgY-Cy2 (Donkey polyclonal)	Gift from Dr.Shaoyu Ge, Stony Brook University NY		IHC (1:1000)
chemical compound	NeuroTrace^™^ 435/455 Blue Fluorescent Nissl Stain	Thermo Fisher	Cat#: N21479	IHC (1:500)
recombinant DNA reagent	pAAV-hSyn-DIO-hM4D(Gi)-mCherry (plasmid)	Addgene	Cat#44362	
recombinant DNA reagent	pV2SGE (plasmid)	This paper	Gift from Dr.Shaoyu Ge, Stony Brook University NY	
recombinant DNA reagent	pAAV-TRE-DIO-oChlEF-mCherry-P2A-tTAH100Y.SV40 (plasmid)	This paper	Deposited to Addgene	Addgene Cat# 169414
recombinant DNA reagent	pAAV-TRE-DIO-hM4Di-mCherry (plasmid)	This paper	Deposited to Addgene	Addgene Cat# 169415
sequence-based reagent	chat_F	IDT	PCR primers	TCTGGCAACTTCGTCGGA
sequenced-based reagent	chat_R	IDT	PCR primers	CTCCTGGGCTGTTACGCAC
sequenced-based reagent	pV2.1-Gene Block 7xTetO-LoxP-Lox2272-tTAH100Y.SV40	IDT	Gene block, custom	
sequenced-based reagent	pV2.2-Gene Block oChlEF-LoxP-Lox2272	IDT	Gene block, custom	
commercial assay or kit	In-Fusion^®^ HD Cloning Plus	Takara/Clontech	Cat#: 638920	
commercial assay or kit	High-Capacity cDNA Reverse Transcription Kit	Applied Biosystems	Cat#: 4368814	
peptide, recombinant protein	T4 DNA Ligase	NEB	Cat#M0202S	
peptide, recombinant protein	Bglll	NEB	Cat#R0144S	
peptide, recombinant protein	AscI	NEB	Cat#R0558S	
peptide, recombinant protein	BamHI-HF	NEB	Cat#R3136S	
peptide, recombinant protein	Pmll	NEB	Cat#R0532S	
peptide, recombinant protein	Phusion^®^ High-Fidelity DNA Polymerase	NEB	Cat#M0530S	
chemical compound, drug	Clozapine	Sigma Aldrich	Cat#C6305–25MG	
chemical compound, drug	Fast Blue	Polysciences Inc.	Cat#17740–1	
other	Mt.Lion Pee	Maine outdoor solutions LLC		
software, algorithm	Prism	GraphPad Software Inc.	RRID:SCR_002798	
software, algorithm	Sigmaplot 12.5	Systat Software Inc.	RRID:SCR_003210	
software, algorithm	OriginPro 9.1	Origin Lab Corporation	RRID:SCR_014212	
software, algorithm	Fiji is just imagej	Fiji	RRID:SCR_002285	
software, algorithm	FreezeFrame v3	Actimetrics	RRID:SCR_014429	
software, algorithm	MATLAB	Mathworks	RRID:SCR_001622	
software, algorithm	Pre-processing Analysis MATLAB Script for FiberPhotometry	Doric		
software, algorithm	ACh sensor analysis MATLAB script	Crouse, Richard B., et al. Elife 9 (2020): e57335.		
